# A Rare Case of Bilateral Achilles Tendon Xanthomas in a Teenager, Successfully Treated with Tendon Sparing Technique

**DOI:** 10.1155/2021/1932763

**Published:** 2021-08-04

**Authors:** G. A. P. K. Appuhamy, B. M. Munasinghe, L. M. Soysa, D. Nelson, P. G. P. Ranmini, W. T. Pavithra, W. M. Sajini, C. D. N. Anthony

**Affiliations:** ^1^Orthopaedic Unit, District General Hospital, Mannar, Sri Lanka; ^2^Department of Anaesthesiology and Intensive Care, District General Hospital, Mannar, Sri Lanka; ^3^Department of Radiology, District General Hospital, Mannar, Sri Lanka

## Abstract

**Background:**

Xanthoma of the Achilles tendon, even though being benign, is a surgically challenging orthopaedic condition. Causality is believed to be due to a pathological error in the metabolism of low-density lipoprotein and their resultant accumulation, as foam cells within the tendon. Tendon xanthomas are often found to accompany heterozygous familial hypercholesterolemia. *Case Presentation*. A 19-year-old girl presented to our institution (a District General Hospital), with soft tissue lumps over posterior aspect of the ankle on both sides for several years. She had noticed a rapid increase in size in recent 3 months and sought medical advice. During investigation, she was diagnosed having bilateral Achilles tendon xanthomas clinically, confirmed by ultrasound scan and magnetic resonance imaging, and familial hypercholesterolemia concomitantly. The former was managed with intralesion subtotal resection where the histology further confirmed the diagnosis. The patient was commenced on statins and followed up while assessing the functional outcome and recurrences up to 2 years, with favourable results.

**Conclusion:**

Subtotal resection of Achilles tendon xanthoma (tendon sparingly) offers cosmetically and functionally acceptable outcomes, with faster recovery and no recurrences over 2 years.

## 1. Background

A xanthoma refers to a benign exogenous mass, visible on tendons, synovium, and subcutaneous tissues. It is an important clinical manifestation of defects in the lipid metabolism and is frequented with familial hypercholesterolemia (FH) [1] and familial coronary artery disease [2]. Different types of xanthomas are identified, categorised as tendinous xanthomas, xanthoma tuberosum, eruptive xanthoma, xanthoma planum, and palmar xanthoma [3]. Out of all, tendinous xanthomas are the commonest type among patients with FH (40%-50% of all patients). These are mainly located within tendons that are close to the skin, preferably the extensor tendons such as Achilles, patella, and extensor hand tendons [3]. Bilateral Achilles tendon involvement is seen in 90% of cases [4].

The pathogenesis of xanthoma is linked with low-density lipoproteins (LDL), derived from the circulation, which accumilate inside the tendons. Subsequently, LDL is converted to oxidized LDL (Ox LDL). The macrophages actively engulf these Ox LDL and form foam cell clusters [5].

Even though imaging studies illustrate typical features of xanthomas in Achilles tendon, the histology is considered as the definitive diagnostic modality, showing clusters of multinucleated giant cells, histiocytes (foamy macrophages), and hemosiderin deposits [6].

A majority of cases are initially asymptomatic yet could become symptomatic when growing in size, resulting in weakness of plantar flexion, difficulty in walking, and cosmetic discontent, requiring surgery [7]. The following case illustrates a successful bilateral xanthoma excision performed in a young South Asian woman, adopting a tendon sparing technique.

## 2. Case Presentation

A 19-year-old South Asian girl presented with lumps over the posterior aspects of both lower limbs, just above the heel for 4 years, and pain over the swelling for last 3 months. The swelling was gradual in onset, initially the size of a small coin and subsequently progressed up to 8 × 6 × 4 cm at the time of presentation. The dull pain over the lumps was abrupt since last 3 months, aggravated during walking, and relieved with rest.

**Figure 1:**
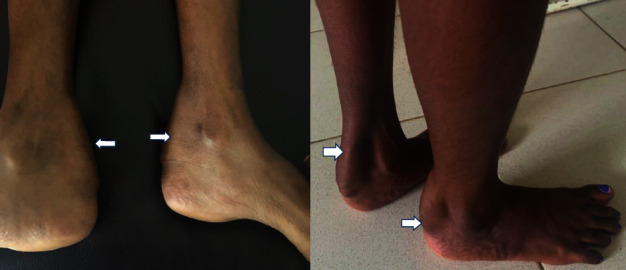
Bilateral tendon xanthomas over Achilles tendon illustrating noticeable swelling (white arrows).

On examination, the lump extended from the lower third of both legs to just above the heels, which were oval in shape, firm in consistency, nonreducible, and nontranslucent. The skin over the lumps was smooth and freely mobile. No features suggestive of inflammation were noted. The mobility of the lumps with the movement of her ankle joint (and restricted horizontal movements) confirmed their origin from Tendo-Achilles. Ankle and subtalar movements were normal and pain free. No muscle wasting or neurovascular deficit was identified (Figure 1).

The patient was concerned about the distorted appearance of her ankle and the difficulty of wearing shoes. Further, the pain over the lumps had been debilitating, especially with long distance walking.

### 2.1. Investigations

Full blood count, erythrocyte sedimentation rate, C-reactive protein level, and thyroid profile were normal. Lipid profile revealed elevated levels. Her parents were simultaneously investigated where both were found to have high cholesterol levels in their lipid profile (Table 1). Electrocardiograms of the trio did not show any features of coronary artery disease.

**Figure 2:**
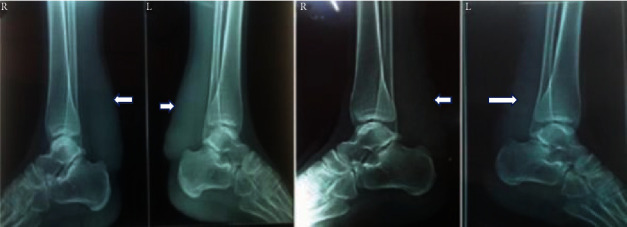
Soft tissue shadow (white arrows) posterior to the ankle in both legs (L: left leg; R: right leg).

X-ray revealed no bony abnormalities but showed thickened, noncalcified soft tissue shadows in the region of the Achilles tendon in the both legs (Figure 2).

**Figure 3:**
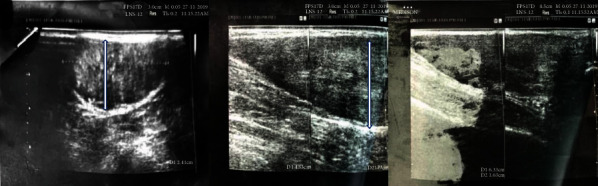
Ultrasound appearance of thickened Achilles tendon with focal hypoechoic areas. White arrows, thickened Achilles tendon.

Ultrasound scan revealed uniformly thickened Achilles tendon with antero-posterior (AP) thickness of >7 mm and multiple hypoechoic focal areas within the tendon (Figure 3).

### 2.2. Magnetic Resonance Imaging (MRI)

The following features were noted in the lower limb MRI study of the patient.

**Figure 4:**
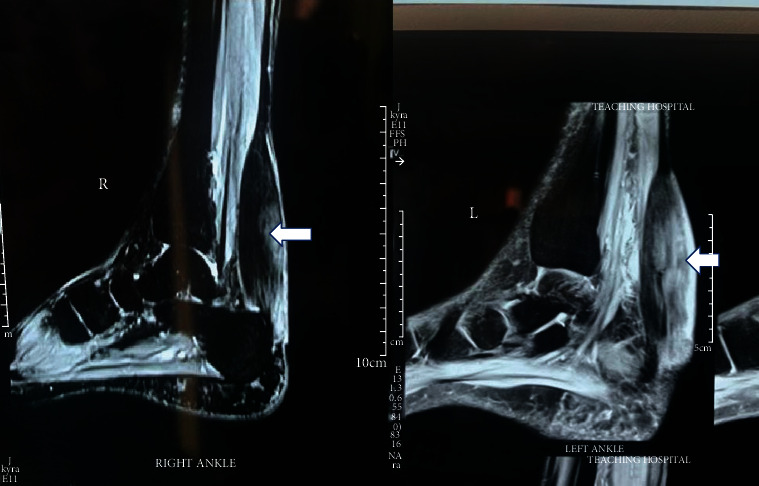
MRI scan of the lower limbs. Sagittal view: right (R) and left (L) tendon xanthomas (white arrows).

Higher T1- and T2-weighted images depicted striated appearance in sagittal sequences due to interposition of xanthoma between tendon fibres while illustrating speckled appearance in axial sequences. The xanthomas were focal with minimal infiltration into tendon tissues (Figure 4).

### 2.3. Surgical Technique

**Figure 5:**
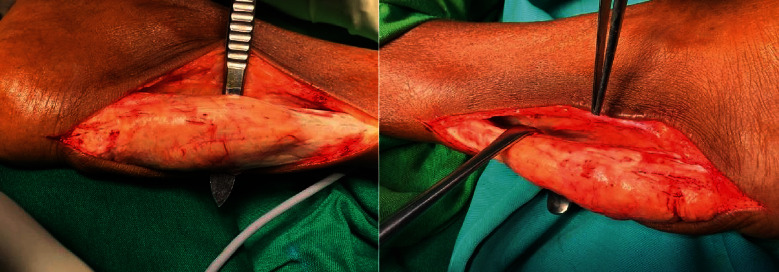
Surgically exposed lump.

Surgery was performed under spinal anaesthesia in a bloodless field. The patient was positioned supine with lower limbs flexed 45° at the hip and knee then fully externally rotated at the hip to visualize the posterior aspect of Achilles tendon. The ankles were held fully dorsi-flexed though out the procedure and operated one at a time. A midline incision was made over the lump, and the tendon was exposed up to tendo-muscular junction, carefully preserving the sural nerve, on the lateral side of the tendon and plantaris tendon (Figure 5).

**Figure 6:**
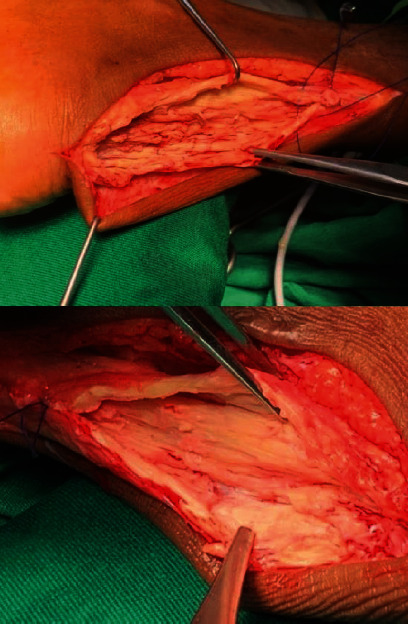
After subtotal resection.

**Figure 7:**
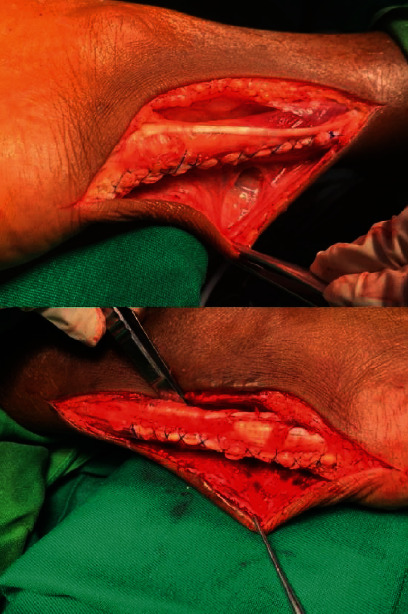
Following the tendon repair.

The tendons were explored via a middle vertical incision, yielding macroscopically intratendinous focal lesion, which was a differentiated xanthoma lesion in contrast to adjacent tendon tissue. Extensive intralesion subtotal resection was performed until the normal surrounding tendon tissue was approached (Figure 6). As we were able to preserve nearly 50% of the tendon tissue, additional tendon enhancements were not required. In both legs, we could preserve plantaris tendon (Figure 7).

The tendon defect was approximated and repaired with Ethibond sutures (Figure 7).

**Figure 8:**
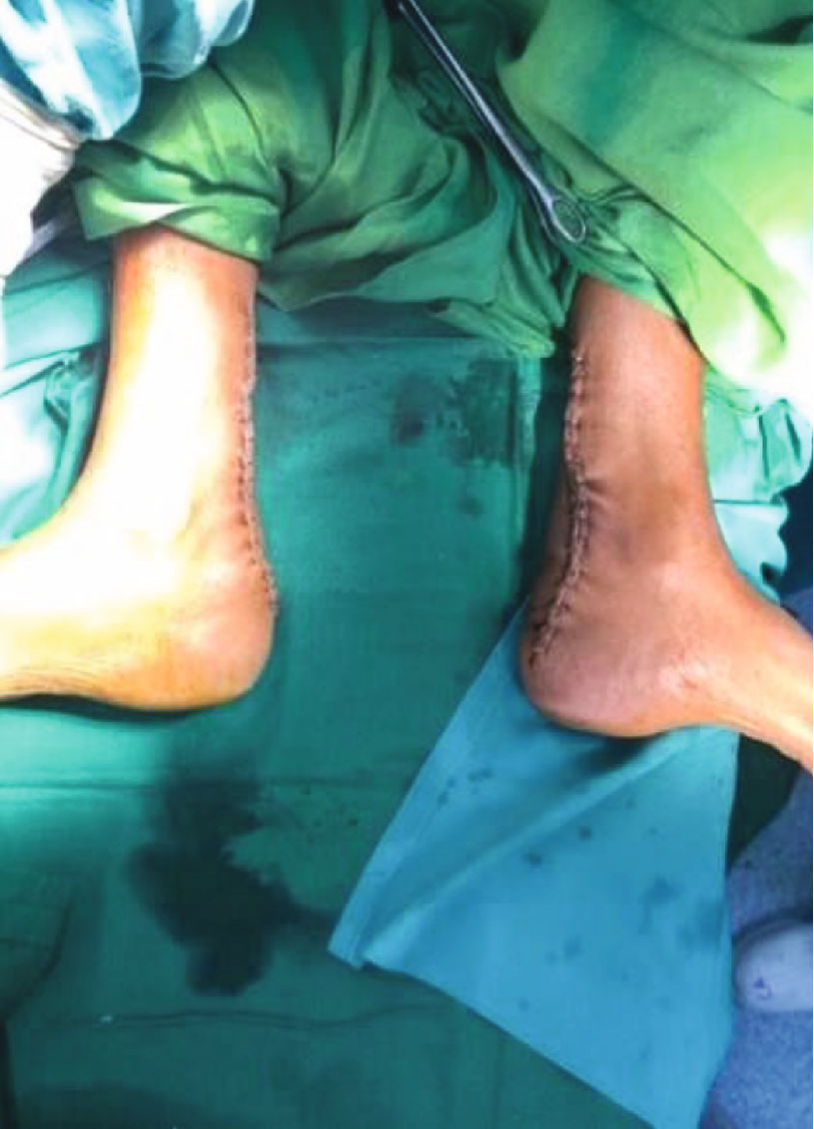
Following skin closure.

The wound closure was done with skin staplers (Figure 8).

### 2.4. Histology

**Figure 9:**
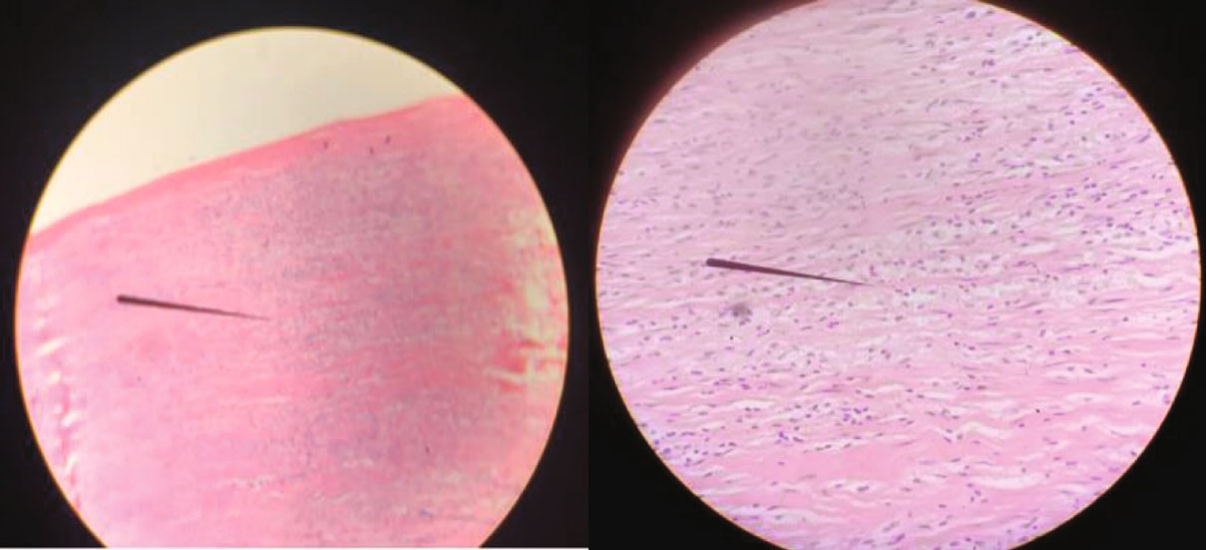
Local concentration of multinucleated giant cells, lipid-laden foamy macrophages along with connective tissue fragments, few inflammatory cells, and cyst macrophages with no malignant cells.

Histology of the tissue biopsy confirmed the diagnosis of xanthoma of the tendon sheath (Figure 9), which consisted of local concentration of multinucleated giant cells, lipid-laden foamy macrophages along with connective tissue fragments, few inflammatory cells, and cyst macrophages with no malignant cells.

### 2.5. Postoperative Care

**Figure 10:**
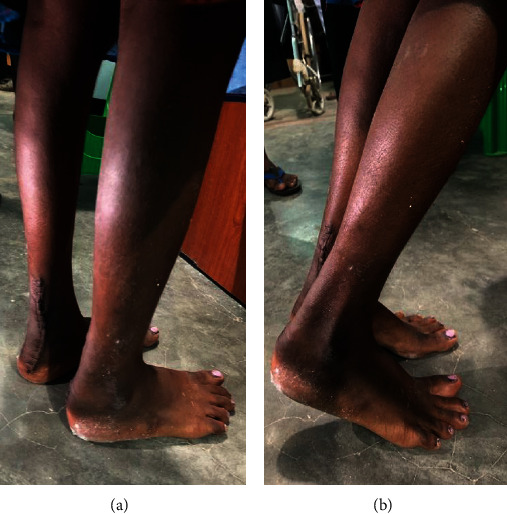
Stance position (a) and standing on toes (b).

The ankles were immobilized with nonweight bearing lower leg plaster of Paris (POP) casts at 20° plantar flexion for about 2 weeks and by wheelchair mobility. Wound stapler removal was done on the 14th post-op day which revealed a healthy surgical site. Active ankle joint movements were commenced, and concurrently walking was initiated utilizing a walker. Six weeks' postsurgery, she was completely independent when walking with ability to stand on toes without discomfort and was able to wear shoes without discomfort (Figure 10).

### 2.6. Medical Management

The patient was started on oral atorvastatin 10 mg daily following referral to the consultant physician, and her lipid profiles were assessed every 3 months.

Her parents were directed to the medical clinic for the treatment of familial hypercholesterolemia.

### 2.7. Follow-Up

**Figure 11:**
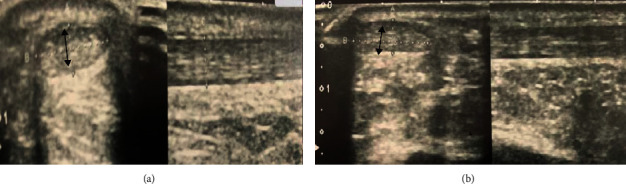
Ultrasound view: (a) right side; (b) left side. AP and longitudinal views illustrating no recurrence of xanthoma in Achilles tendon bilaterally (black arrow).

She was followed up every 2 months up to 2 years in orthopaedic clinic of our institution. She had no recurring symptoms or clinical evidence of recurrence. Ultrasound scan assessments were performed at 1-year and 2-year visits which denoted scar tissue in the absence of recurrence (AP thickness was <4 mm on both Achilles tendons) (Figure 11). Cholesterol level was controlled below the upper limit with medical treatment and was continued.

## 3. Discussion

The Achilles tendon xanthoma is generally a benign albeit uncommon occurrence, which is associated with elevated cholesterol levels [8] and coronary artery disease [2]. The familial hypercholesterolemia with an autosomal dominant inheritance is characterized by an elevated LDL-cholesterol and tendon xanthomas.

In this particular patient, serum total cholesterol was slightly elevated while the levels were significantly above the upper limit in her parents who were previously healthy.

The Achilles tendon xanthomas are commonly bilateral and often asymptomatic. In the natural history of xanthomas, the initiation is as a small lesion which subsequently increases in size, with associated symptoms such as local pain, disfiguration, and pain on ambulation.

Surgical treatment of Achilles tendon xanthomas is challenging and is usually limited to those who have severe disfigurement, pain, or difficulty with mobility due to the mass. Our patient was at the symptomatic stage, warranting surgical intervention.

The literature illustrates mainly two surgical techniques as opposed to conservative care.

1. Complete xanthoma excision and reconstruction of the defect with tendon grafts [9]
2. Subtotal intralesion resection [10]

It is debatable which is more superior; however, mainstream evidence points out that the complete resection with tendon reconstruction leads to less recurrence rates [11], despite increased morbidity during the postoperative period. As summarized by Huang et al., there are several procedures for Achilles tendon reconstruction, which commonly utilize autogenous tendon grafts such as the flexor hallucis longus (FHL) [12] or peroneus brevis.

On the other hand, subtotal resection leads to expedited recovery with early return to work even though the recurrence rate is comparatively higher [13]. Interestingly, few studies have shown reduced recurrence rates even with subtotal resection [2]. If we can preserve the function of the Achilles tendon and other tendons (FHL and peroneus brevis) with less complications and faster recovery, subtotal resection is recommended.

In our case, the patient requested both limbs to be operated together, having to undergo single surgery. According to the ultrasound and MRI studies, her xanthomas were focal in both tendons without gross infiltration of adjacent tissue [14]. Considering all these facts and following a detailed discussion with the patient and her parents about possible outcomes, we performed an extensive subtotal resection of intralesion mass but decided to qualify the procedure as “tendon sparing” (without tendon graft enhancement) as at least 50% of the cross-section of the tendon was salvaged [10]. We believe that this technique is less invasive which results in faster recovery. We used staplers for skin closure as we already had favourable outcomes in our practice using staplers in ankle surgeries for nondiabetic young patients. In addition, literature provides enough supportive evidence for the use of staplers for skin closure following ankle surgeries [15, 16].

Notably, our patient started assisted weight bearing after 2 weeks even though many reconstructive procedures reported significantly longer nonweight bearing periods. Our patient was a completely independent walker by 6 weeks.

In spite quoted to have a higher recurrence rate in vast majority of the literature [2], our patient was free of recurrence or symptoms even at 2 years.

We regularly followed the patient up until 2 years, to assess for recurrence and clinical symptoms, with encouraging results. We utilized ultrasonic imaging in follow-up process as MRI was not freely available in local setup.

## 4. Conclusion

Intralesion subtotal resection is an acceptable solution for symptomatic Achilles tendon xanthomas, with minimal postoperative complications, faster recovery, and prompt return to normal activities.

## Figures and Tables

**Table 1:** Comparison of lipid profile between patient and parents.

	Total cholesterol	Triglyceride	HDL	LDL	VLDL	Chol:HDL
Patient	217	110.8	71.9	103	22.2	2.8
Father	202.1	118	51.8	162	23.1	3.7
Mother	228.2	101.7	53.4	154.5	20.3	4.3
Reference range (mg/dl)	<200	<150	>50	<100	<30	3.5

## Data Availability

Data supporting the results of the manuscript are included within the manuscript.
